# Anxiolytic, antioxidant, and neuroprotective effects of goji berry polysaccharides in ovariectomized rats: experimental evidence from behavioral, biochemical, and immunohistochemical analyses

**DOI:** 10.3906/biy-2003-8

**Published:** 2020-10-13

**Authors:** Fatma PEHLİVAN KARAKAŞ, Hamit COŞKUN, Hayriye SOYTÜRK, Bihter Gökçe BOZAT

**Affiliations:** 1 Department of Biology, Faculty of Science and Art, Bolu Abant İzzet Baysal University, Bolu Turkey; 2 Department of Psychology, Faculty of Science and Art, Bolu Abant İzzet Baysal University, Bolu Turkey; 3 Department of Poultry Science andTechnology, Faculty of Agriculture and Natural Science, Bolu Abant İzzet Baysal University, Bolu Turkey; 4 Disciplinary Neuroscience, Health Sciences Institute, Bolu Abant İzzet Baysal University, Bolu Turkey

**Keywords:** Lycium barbarum L., pharmacological activity, antioxidant enzymes, neurotransmitters, serotonin, BDNF, ELISA, menopause

## Abstract

Recent studies have indicated that polysaccharides, the main component of the *Lycium barbarum* L. fruit, have beneficial effects (e.g., anxiolytic, antioxidant, and neuroprotective) on humans and rodents. However, the effects of different dosages of such polysaccharides on ovariectomized rats and their underlying mechanisms in the brain have not been evaluated in the literature. Here, we aimed to evaluate the effects of the high and low doses of polysaccharides obtained from *Lycium barbarum* fruits (HD-LBP and LD-LBP, respectively) on anxious behaviors via behavioral (using the OFT and EPM), biochemical (using ELISA), and immunohistochemical (using immunohistochemical staining) measures in detail. Two weeks after ovariectomy, the rats were randomly assigned to either the treatment conditions [control (DW, 3 mL/kg, p.o., per day), LD-LBP (20 mg/kg, 3 mL/kg, p.o., per day), HD-LBP (200 mg/kg, 3 mL/kg, p.o., per day), 17 β-ES (1 mg/kg, 3 mL/kg, p.o., per day), DZ(1 mg/kg, 3 mL/kg, p.o., per day)] or operation type [SHAM (pseudo-ovariectomized) and OVX (ovariectomized)]. The treatments were applied for 30 consecutive days, and then serum and brain tissue samples of all rats were collected. Biochemical (SOD, CAT, GPX, MDA, and 17 β-ES) and immunohistochemical (BDNF, SER, and apoptosis) analyses of the samples were performed as well. The rats administered HD-LBP and LD-LBP were less anxious than the control groups. The HD-LBP–treated rats had high levels of SOD and low levels of MDA in their serum samples. Moreover, HD-LBP and drug-treated groups had a high number of SER receptors and BDNF-positive cells and a low number of TUNEL-positive cells in their hippocampal brain tissues. The HD-LBP treatments decrease anxious behavior by increasing antioxidant enzyme activities, hippocampal SER and BDNF neurotransmitter levels and decreasing the TUNEL-positive cell count of ovariectomized rats. Given these findings, we suggest that menopause-induced symptoms of anxiety can be reduced by polysaccharides obtained from goji berry fruits, and that these findings will be beneficial for the production studies of natural herbal-origin antianxiety (anxiolytic) drugs in the future.

## 1. Introduction

*Lycium barbarum* Linnaeus, which is also known as goji berry, wolfberry, super fruit, *Lycium* fruits or *Fructus lycii* (Yang et al., 2018), belongs to the genus *Lycium* of the family Solanaceae (Zheng et al., 2015). The fruits have been used in herbal medicine and health food for thousands of years in China, Southeast Asia, Europe, and North America (Potterat, 2010; Bilgic et al., 2015; Zheng et al., 2015). Consistent with its widespread use, some recent studies have provided evidence for *L. barbarum* ’s beneficial effects, such as anxiolytic, protection of eyesight, immunity, and prevention of several metabolic disturbances including diabetes hyperlipidemia (Amagase and Farnsworth, 2011; Pehlivan Karakas et al., 2016). These beneficial effects of goji berry for humans could be attributed to its dietary constituents, such as flavonoids, polysaccharides, phenolics, vitamins, and carotenoids (Pehlivan Karakas, 2020). Among them are polysaccharides, the main component of the *L. barbarum* fruit (Gaoet al., 2017), which has recently received special research attention. For instance, several animal studies have indicated that *Lycium barbarum* polysaccharides (LBP) have ocular neuroprotective, antioxidant, immunomodulator, hepatic protective, and antitumor effects in animals (Chang and So, 2008; Cheng and Kong, 2011). LBP has been shown to alleviate neuronal injury and obstruct lactate dehydrogenase release (Rui et al., 2012); LBP can regulate phosphatidylinositol 3-kinase/Akt pathway/endothelial nitric oxide synthase (PI3 K/Akt/eNOS) signaling pathways in ovariectomized rat myocardium, thus exerting an antioxidative effect (Ning et al., 2016). Moreover, previous research has shown that LBP enhances superoxide dismutase (SOD) and glutathione peroxidase (GPX) enzyme activities; however, it decreased their malondialdehyde (MDA) content (Teng et al., 2013). Furthermore, LBP deactivates caspase-3; thus, LBP may have applications in the treatment of neuronal apoptosis induced neurodegenerative diseases (Teng et al., 2013). Recent research has provided evidence for many beneficial effects of LBP on rodents, such as antidepressant properties (Zhang et al., 2012). Despite these findings in normal animals, the effects of LBP on the anxiety of ovariectomized rats, as well as analyzing the effects of different dosages on the activity of enzymes and their underlying mechanisms in the rat brain, have not been investigated yet in a single research paradigm.

Steroid hormones can have significant effects on the life of neurons and glial cells and regulation of brain functions (Rettberg et al., 2014; Brinton et al., 2015). In particular, changing estrogen concentrations in the central nervous system after menopause affect some brain region functions such as the amygdala, prefrontal cortex, and hippocampus (Zhang et al., 2019). Physiological alterations in the brain regions affect the emotional, cognitive, and behavioral aspects of women’s lives, causing issues such as anxiety and depression disorders and memory impairment (Rettberg et al., 2014; Berent-Spillsona et al., 2016). Declining estrogen levels after menopause/ovariectomy may cause an accumulation of oxidative stress in blood serum and thus change antioxidant enzyme concentrations and cause behavioral disruptions. Brain tissue in particular demands more oxygen and has a restricted level of antioxidant capacity; thus, it is sensitive to oxidative stress induced by reactive oxygen species (ROS) (Halliwell and Gutteridge, 1985; Asha Devi et al., 2011; Belviranlı et al., 2013). Accumulation of oxidative stress in the brain tissue results in many neurodegenerative disorders due to damaged proteins, lipids, and nucleic acids. Moreover, oxidative stress causes damage and apoptosis of neurons because it especially disrupts proteins and deoxyribonucleic acid (DNA) in the brain (Valvassori et al., 2015). Given such plausible effects of ovariectomy on oxidative stress, it is important to discover new techniques or treatments such as different dosages of LBP for eliminating this stress level and to investigate its effects on the parameters of oxidative stress or related enzyme activities and neurotransmitters in the nervous system and brain.

Serotonin, an important monoamine neurotransmitter, is produced in the raphe nuclei by tryptophanhydroxylase 2. It also plays an important role in mood disorders (Banerjee and Poddar, 2016; Dornellas et al., 2018). It is predominantly found in the somatodendritic region of the hippocampus and medial temporal lobe memory system (Pithia et al., 2016). Approximately 15 types of serotonin receptor (SER) have been reported, and they are connected to 7 SER families (Kroeze et al., 2002; Banerjee and Poddar, 2016). Decreased estrogen levels after menopause have been found to affect the levels of serotonin and dopamine (Shaif et al., 2018). Estrogen in particular regulates the levels of SER via enhancing the expression of tryptophan hydroxylase (Han et al., 2018). Brain-derived neurotrophic factor (BDNF) promotes neuronal proliferation, survival, and maintenance of neuron structure and function (Li et al., 2018). More recent studies have found that estrogen also regulates BDNF expression in the hippocampus and cerebral cortex (Miranda et al., 2019) and plays a neuroprotective role in the hippocampus via increasing BDNF levels (Fatemi et al., 2019). Moreover, recent research has also shown that ovariectomy decreases BDNF levels, which contributes to cognitive impairment (Zhang et al., 2019).

The purpose of this study was to investigate the anxiolytic effect of a low dose of LBP (LD-LPB) and a high dose of LBP (HD-LBP) on the anxious behavior of ovariectomized female rats, as well as the biochemical and immunohistochemical mechanisms behind the anxiousbehaviors. Given these considerations, we hypothesized that HD-LBP would decrease the anxiety level of ovariectomized female rats, as our previous study demonstrated that methanol extraction of *L. barbarum* fruit decreases the anxiety level of rats (Pehlivan Karakaş et al., 2016). However, our previous research did not include ovariectomized female rats or establish the pure effect of extracted polysaccharides of gojiberry. Furthermore, we aimed to investigate whether the anxiolytic effect of LBP would increase the levels of antioxidant enzymes and whether it would increase SER and BDNF receptors in the brain due to this increase. We hypothesized that the antioxidant enzyme levels would increase in the blood serum of the LBP-treated groups and thus the SER and BDNF receptor numbers would increase in brain tissue. For the first time, the effects of polysaccharides derived from goji berry fruits on anxiety in ovariectomized rats have been examined in terms of behavioral, biochemical, and immunohistochemical aspects in the literature.

## 2. Materials and methods

### 2.1. Animals

Female Wistar albino rats 3–4 months of age and with a body weight of 180–250 g were used. Rats were purchased from Bolu Abant İzzet Baysal University (BAIBU) Experimental Animal Application and Research Center. A total of 100 female rats were used in this study. Seven days before the start of the experiments, female rats were housed individually in plastic cages (40 × 50 × 20 cm) with a temperature-controlled environment (22 ± 2 °C) and a reversed light/dark (12/12 h) cycle (lights on at 8:00, and lights off at 20:00). Food and water were given ad libitum until the time of the experiments. All applications were performed between 13:00 and 17:00 to avoid potential circadian alteration (Russo et al., 2013; Citraro et al., 2015). Animals fasted except for water for 12 h before gavage. All behavioral procedures were performed during the light cycle. The animal experimental procedures were carried out following the Animal Scientific procedure and approved by the Institutional Animal Care and Use Committee. The study was carried out according to the guidelines of the Ethics Committee of BAIBU; in addition, all of the treatments complied with the recommendations of the Declaration of Helsinki (registration number: 2014/35). All efforts were made to diminish animal pain and to minimize the number of animals used (Pic-Taylor et al., 2015). 

### 2.2. Surgical procedure

Ovariectomy (OVX) or false surgery (SHAM) operations were performed on 3–4 month-old female Wistar rats. The female rats were anesthetized by intraperitoneal injection with a mixture of Ketamine/Xylazine (4:1, 0.25 mL i.p.). The OVX was applied over a midline abdominal incision (2 cm in length) in the linea alba, with cauterization, crushing, and ligating of the fallopian tubes; ovaries were gently bilaterally removed by cutting above the clamped area. The rats in the sham-operated group were exposed to the same OVX process except for ovary removal (Bozdoğan et al., 2018). After 10 days of recovery from their ovariectomies, the rats were exposed to gavage application for 30 consecutive days. All behavioral tests commenced after the gavage.

### 2.3. Preparation of L. barbarum polysaccharides

*Lycium barbarum* polysaccharide was prepared as described in previous reports (Zhang et al., 2015; Wang et al., 2016). The *L. barbarum* fruits were purchased from Gojiform®, Turkey. Two hundred grams of dried fruits were crushed and extracted 2 times with 600 mL of chloroform:methanol (2:1) solution at 80 °C. The mixture was then filtrated, and 600 mL of 80% ethanol was added twice to remove some colored material; each extraction lasted 2 h. The second mixture was filtrated and the dry residuals were extracted with hot water (80°C) 3 times (for 2 h each time). Reflux extraction was conducted with 95% ethanol for precipitation at 4 °C. The precipitation was collected by centrifugation (3400 rpm, 15 min) 12 h after the process and washed with pure ethanol and acetone. After the washing, the precipitation was dried under reduced pressure. The dried LBP used in this in vivo study comprised 7 kinds of monosaccharides (i.e. arabinose, galactose, glucose, rhamnose, mannose, xylose, and galacturonic acid) (Amagase and Farnsworth, 2011).

### 2.4. Experimental groups

One hundred female Wistar rats were randomly assigned to the 2 main groups: OVX and SHAM. These groups were then subdivided into 5 treatment groups (n = 10): HD-LBP, LD-LBP, 17 beta-estradiol *(* 17 *β* -ES), diazepam (DZ),and distilled water (DW). All of the experimental groups were treated orally by gavage (3 mL/kg body weight) once a day during the 30-day treatment.

HD-LBP (200 mg/kg perday), LD-LBP (20 mg/kg perday), 17 *β* -ES (1 mg/kg perday), DZ (1 mg/kg perday), and DW (3 mL/kg perday) were given to both OVX and SHAM groups and administered orally for 4 weeks, based on previous reports (Cui et al., 2010; Wu et al., 2010; Bradley et al., 2011; Duan and Sun, 2012). At the end of all treatments, behavioral functions were investigated after the last application using the open field test (OFT) and elevated plus-maze test (EPM) for the measurement of anxious behaviors. After the rats were euthanized by decapitation, blood samples and brain tissue specimens were collected. 

### 2.5. Behavioral tests

#### 2.5.1. Open field test (OFT)

The open field test was performed after the last day of treatment. A single rat was placed in the center of a black Plexiglas square measuring 80 cm in length × 80 cm in width × 40 cm in height. The subject explored the environment for 15 min in the training session. After the training session, the rat was exposed to the test for 5 min in the test session. During the test session, time spent in the center arena and edges was monitored by a video camera (Gkb CC-28905S, Commat Ltd. Şti., Ankara, Turkey) and recorded with a videotape interfaced with an EthoVision video tracking system (NoldusEthovision, Version 6, Wageningen, Netherlands; Commat Ltd. Şti., Ankara, Turkey). During the test session, the frequency of the entry to the center area, the number of center entries, and time spent in the center area was recorded, and recorded data were calculated by the program. Decreased activity indicates an increased level of anxiety. Mean velocity and total distance moved in the test session were also recorded and analyzed by the program (Russo et al., 2013).

#### 2.5.2. Elevated plus maze test (EPM)

The elevated plus maze test is the most frequently utilized test for evaluating anxious behavioral states. It is especially sensitive to anxiety-reducing drugs such as anxiolytic agents (Walf and Frye, 2007). The structure of the EPM apparatus is black in color and is composed of 2 open arms which are crossed by 2 closed arms of equal size (55 × 10 cm) with 41-cm–high walls as previously described (Guimaraes et al., 2015). The maze was elevated to a height of 55 cm above floor level. Rats of each group were placed individually in the central area of the EPM to explore for 5 min. Time spent on open arms, time spent on closed arms, and the number of open and closed arms entries were recorded by the EthoVision video tracking system. After each test, the apparatus was cleaned with 70% ethanol. 

### 2.6. Blood and tissue sample collecting 

All animals were anesthetized using ketamine hydrochloride (50 mg/kg body weight) and xylazine (10 mg/kg body weight) and sacrificed immediately after the behavioral test. The systemic blood was collected using the cardiac puncture method. The samples were centrifuged to obtain the serum to analyze the 17 β-ES and oxidative stress biomarker estimations such as SOD, catalase (CAT), GPX, and MDA using a commercial kit (Sunred) and a spectrophotometer (Da Silva et al., 2014). The 17 β-ES, SOD, CAT activity, and MDA content in the rats’ serum were measured according to the kit manufacturer’s protocol (Winching, Nanjing, China) as described previously, using an enzyme-linked immunosorbent assay (ELISA) kit (Sunred) (Patki et al., 2013). After performing the OFT and EPM tests, 5rats from each group were randomly chosen for histological studies. After anesthesia with an overdose of ketamine–xylazine, perfusion–fixation was applied. The skulls of the animals were opened, the brain tissues were removed, placed in 10% neutral formaldehyde, and were given code numbers.

### 2.7. Immunohistochemistry

**Figure 1:**
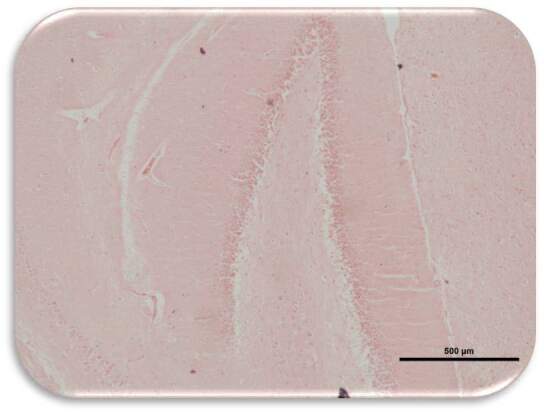
Hemotoxylin and eosin (H&E) staining of hippocampus area of female rat.

After 72 h, the brain tissues were divided into 2 lobes and each right lobe of brains were chosen to be analyzed. The tissues were processed for analyses. First, the tissues were washed and dehydrated in an incremental alcohol series, and then cleared entirely in a xylene series. the tissues were then immersed in liquid paraffin and embedded in paraffin blocks. The blocks were cut 5 μm thick using a microtome (Leica RM2125RT, Wetzlar, Germany) and the acquired sections were brought from deparaffinization to the water and stained with hematoxylin and eosin (H&E) for histopathological investigation with a conventional photomicroscope (Olympus BX51, Tokyo, Japan), then photographed using a camera system (Olympus DP72) by 2 experienced independent histologists (Cevik et al., 2015). A prepared histological sample from a female rat with H&E staining of the hippocampus area is shown in Figure 1. Histopathologic changes were examined in 10 randomly selected hippocampus areas of 5 sections for each group. Evaluations and scoring of the histopathologic changes in the hippocampus were performed for levels of SER, BDNF, and neuronal death (apoptosis) (Selli et al., 2016). The levels of SER, BDNF, and apoptosis in hippocampal tissue were measured by immunohistochemical staining and terminal deoxynucleotidyl transferase dUTP nick end labeling (TUNEL) staining methods (Millipore) according to the manufacturer’s instructions. Immunohistochemistry SER and BDNF staining were identified as either negative or positive. Immunohistochemical positive staining was defined as the detection of brown chromogen on the edge of the hematoxylin-stained cell nucleus, distributed within the cytoplasm or in the membrane, and evaluated as previously described (Musumeci et al., 2015b). Digital pictures were taken with a digital camera (Canon, Tokyo, Japan) at 20×, 40×, and 63.5× magnifications.

### 2.8. Statistical analysis

Data were analyzed grouped by 2 operations (SHAM and OVX) × 5 treatments (LD-LBP, HD-LBP, DZ, 17 β-ES, and DW) using ANOVA analysis, with Tukey and Bonferroni tests for post hoc multiple comparisons. The data are presented as mean ± SEM. A P-value < 0.05 was considered to be statistically significant (Liu et al., 2015).

## 3. Results

### 3.1. Behavioral tests

#### 3.1.1. Open field measurements

**Figure 2:**
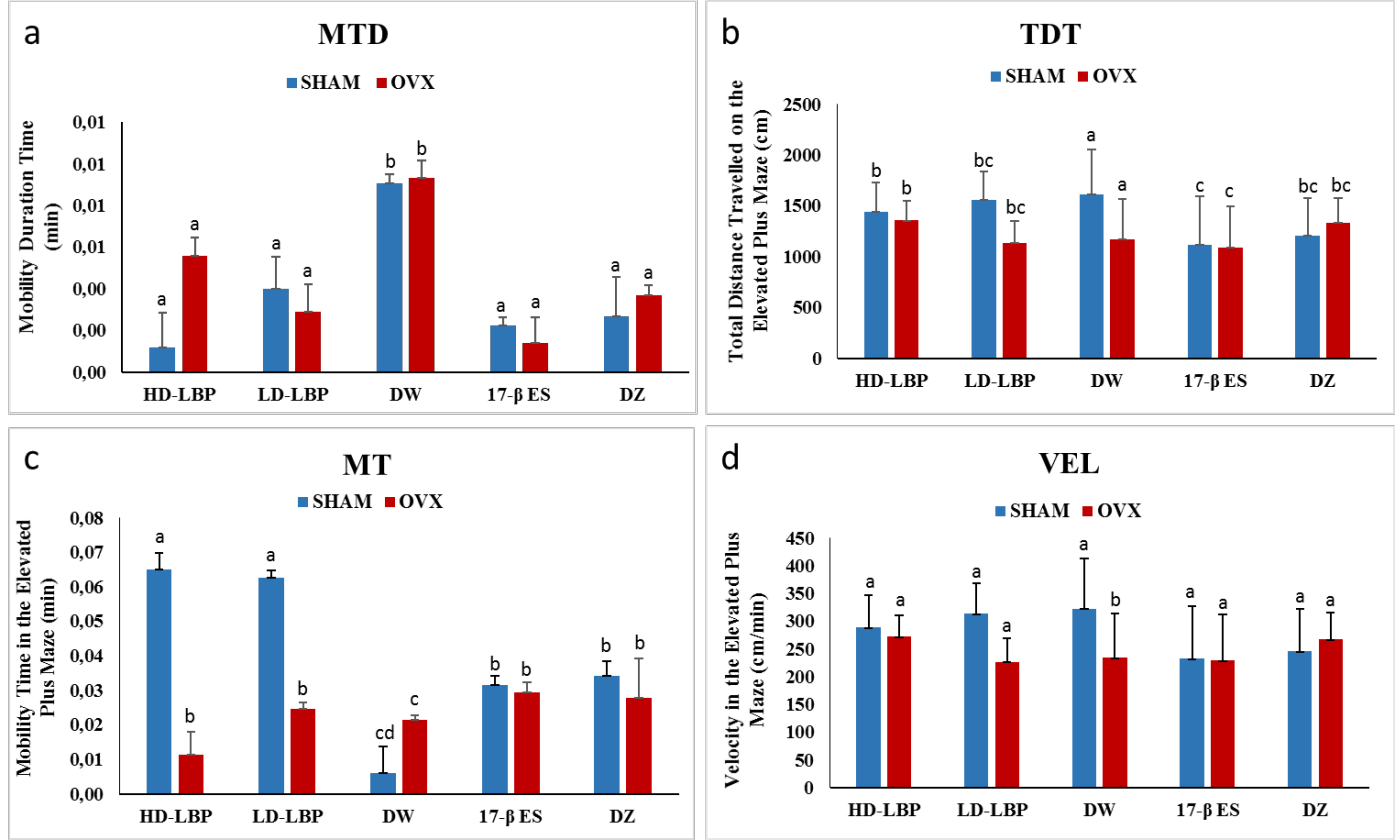
(a) Mean of the mobility duration time (MDT) of center in the open field, (b) mean of the total distance travelled (TDT), (c) mean of the mobility time (MT) in the elevated plus maze, and (d) mean of the velocity (VEL) in elevated plus maze. Mean-values with the same letters within vertical columns are significantly different (P < 0.05).

In this paper, nonsignificant findings were not reported due to limited space. Operation condition had a significant effect on time spent at the edge of the open field [F (1,81) = 17.47, P = 0.0001, **ɳ** p2= 0.18]. The subjects in the SHAM groups spent less time at the edge of the open field than those in the OVX groups (MSHAM = 4.77 < MOVX = 4.91). No significant findings were reported in terms of operation and treatment condition or the interaction between these variables. When all the groups were studied, treatment had significant effect on mobility time on the open field [F (4,78) = 5.33, P = 0.001, **ɳ** p2= 0.21]. The Bonferroni test showed that the subjects in the DW condition were more mobile than those in the other treatment conditions, indicating the lowest anxiety level in the DW group (MDW = 0.009 > MHD-LBP = 0.003, MLD-LBP = 0.003, M17-β ES = 0.002, MDZ = 0.003) (Figure 2a). In sum, the presence of ovariectomy in subjects with DW and LD-LBP conditions reduced open-field anxiety. This finding indicates that HD-LBP may be as effective as 17 β-ES and DZ on open-field anxiety. 

#### 3.1.2. Elevated plus maze measurement

The main effect shown of the operation was a significant effect on total distance traveled on the elevated plus maze [F (1,78) = 5.26, P = 0.02, **ɳ** p2= 0.06]. The statistical analysis test showed that the subjects in the SHAM group traveled greater distances than those in the OVX group (MSHAM = 1389.15 > MOVX= 1216.40) (Figure 2b). This means that subjects in the sham-operation condition were less anxious than those in the OVX condition. Despite the fact that an interaction effect between treatment and operation did not reach conventional significance level [F (4, 78) = 2.10, P > 0.09, **ɳ** p2= 0.10], the subjects in the DW-treated groups in the sham-operated condition spent more time in open arms than those in the OVX condition (MDW-SHAM = 1.25 > MDW-OVX = 0.80). However, subjects in HD-LBP-treated, 17 β-ES-treated, and DZ-treated groups in the sham-operated condition spent more time in the open arms than those in the OVX condition (MHD-LBP-SHAM = 0.69, MHD-LBP-OVX = 0.91; M17 β-ES-SHAM = 0.68, M17 β-ES-OVX = 0.60; MDZ-SHAM = 0.74, MDZ-OVX = 0.93). The main effect of treatment was significant onmobility [F (4, 73) = 4.69, P = 0.002, **ɳ** p2= 0.20]. The Bonferroni test showed that subjects in the HD-LBP- and LD-LBP-treated groups (MHD-LBP = 0.04 and MLD-LBP = 0.04) were more mobile than those in the DW-treated groups (MDW = 0.01). This means that subjects in the HD-LBP- and LD-LBP-treated groups were less anxious than those in the DW-treated groups.The operation had a significant effect on mobility duration in the elevated plus maze [F (1, 73) = 12.20, P = 0.001, **ɳ** p2= 0.14]. The subjects in the sham-operated groups were more mobile than the subjects in the OVX groups (MSHAM= 0.04 > MOVX= 0.02). An interaction effect between treatment and operation was significant [F (4, 73) = 6.96, P = 0.0001, **ɳ** p2= 0.28]. The subjects in the HD-LBP- and LD-LBP-treated groups in sham-operated condition were more mobile than those in OVX condition (MHD-LBP-SHAM = 0.06, MHD-LBP-OVX = 0.01; MLD-LBP-SHAM = 0.06, MLD-LBP-OVX = 0.02), indicating the lowest anxiety levels in HD-LPB and LD-LBP conditions. In the 17 β-ES-and DZ-treated groups, there was little difference between those in the sham and OVX conditions (M17 β-ES-SHAM = 0.03, M17 β-ES-OVX = 0.03; MDZ-SHAM = 0.03, MDZ-OVX = 0.03) (Figure 2c). This means that 17 β-ES and DZ treatment were more positively effective than LBP treatments in the ovariectomy-induced anxious behavior of the rats. The operation had a significant effect on the velocity [F (1, 78) = 5.22, P = 0.02, **ɳ** p2= 0.06]. The subjects in the sham-operated condition were faster than the subjects in the OVX groups (MSHAM = 280.70 > MOVX = 246.00). This means that OVX operation caused the anxious behavior in the female rats. An interaction effect between treatment and operation was not significant [F (4, 78) = 2.21, P > 0.07, **ɳ** p2 = 0.10]. Although subjects in DW-treated groups in the sham-operated condition were faster than those in the OVX condition (MDW-SHAM = 322.72 > MDW-OVX = 234.21), velocity of subjects in the HD-LBP-treated, 17 β-ES-treated, and DZ-treated groups in the sham-operated condition was close to that of subjects in the OVX condition (MHD-LBP-SHAM = 289.25, MHD-LBP-OVX = 272.63; M17 β-ES-SHAM = 232.42, M17 β-ES-OVX = 228.97; MDZ-SHAM = 245.66, MDZ-OVX = 267.13) (Figure 2d).

### 3.2. Biochemical analysis

#### 3.2.1. Antioxidant enzyme levels

##### 3.2.1.1. SOD levels

**Figure 3:**
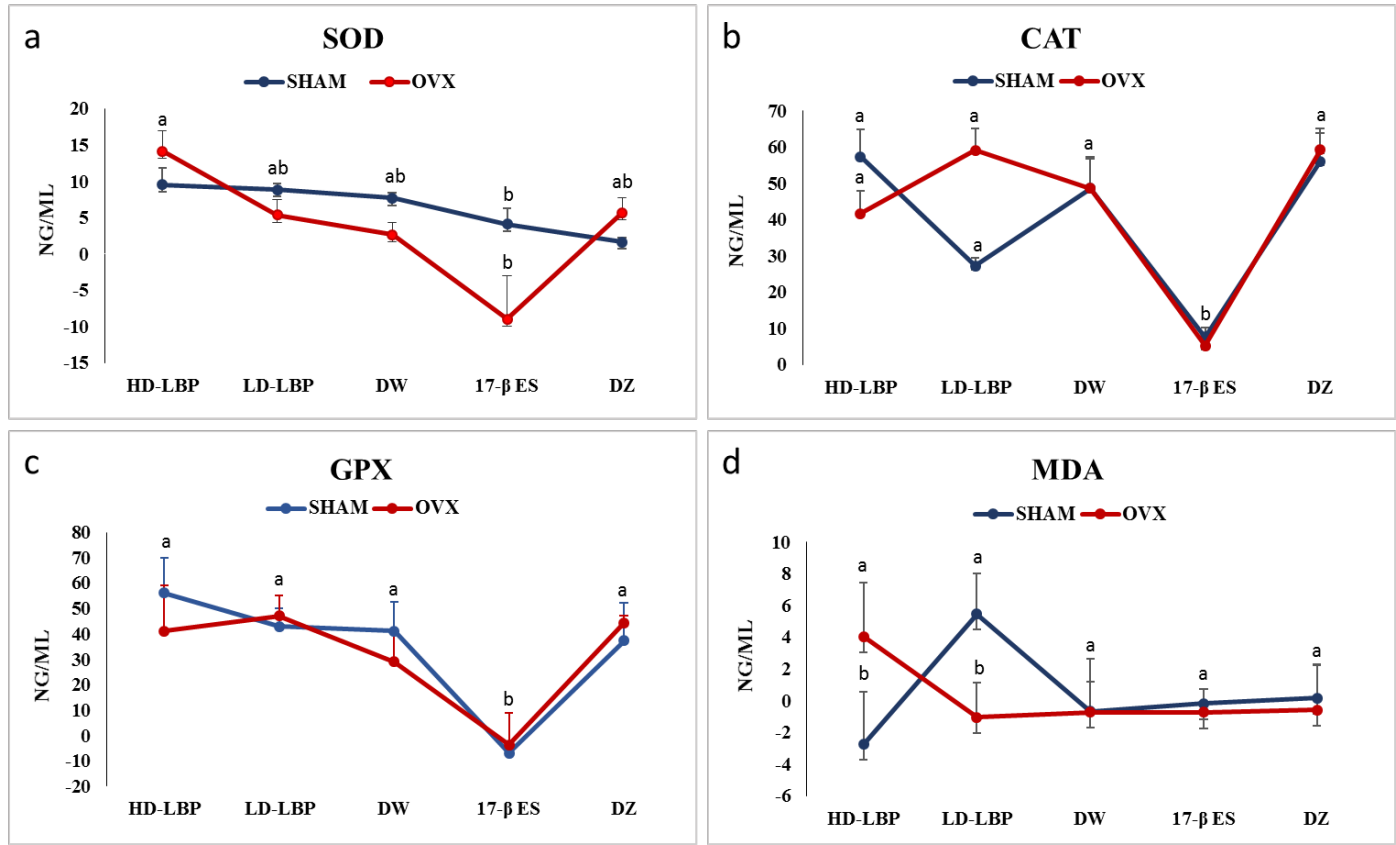
Mean value of SOD (a), CAT (b), GPX (c), and MDA (d) enzyme levels. Mean-values with the same letters within vertical columns are significantly different (P < 0.05).

The main effect of treatment was significant on SOD enzyme activity in the serum [F (4, 41) = 4.18, P = 0.006, **ɳ** p2= 0.29]. The serum of subjects in the HD-LBP-treated groups had higher amounts of SOD enzyme activity than the serum of those in the 17 β-ES-treated groups (MHD-LBP = 11.85 > M17 β-ES = –2.45). An interaction effect between treatment and operation was not significant [F (4, 41) = 2.03, P> 0.10, **ɳ** p2 = 0.11]. Blood serum of the subjects in the HD-LBP- and DZ-treated groups in the OVX condition had a higher level of SOD enzyme activity than those in the sham-operated condition (MHD-LBP-SHAM = 9.57 < MHD-LBP-OVX = 14.13; MDZ-SHAM = 1.65 < MDZ-OVX = 5.68). On the other hand, the serum of the subjects in the other treatment groups in the OVX condition had lower amounts of SOD enzyme activity than those in the sham-operated condition (MLD-LBP-SHAM = 8.87 > MLD-LBP-OVX = 5.37; MDW-SHAM = 7.72 > MDW-OVX = 2.61; M17 β-ES-SHAM = 4.08 > M17 β-ES-OVX =-9.0) (Figure 3a).

##### 3.2.1.2. CAT levels

Treatment had a significant effect on CAT enzyme activity in the blood serum [F(4, 41) = 11.31, P = 0.0001, **ɳ** p2 = 0.53]. Blood serum of subjects in the 17 β-ES-treated groups had lower levels of CAT enzyme activity than those in the other treated groups (M17 β-ES = 6.45 < MLD-LBP = 43.15 < MDW = 48.56 < MHD-LBP = 49.52 < MDZ = 57.63) (Figure 3b).

##### 3.2.1.3. GPX levels

Treatment had a significant effect on GPX enzyme activity in the blood serum [F(4, 41) = 43.89, P = 0.0001, **ɳ** p2 = 0.81]. Blood serum of subjects in the 17 β-ES-treated groups had lower levels of GPX enzyme activity than those in the other treatment groups (M17 β-ES = –5.25 < MDW = 11.85 < MDZ = 40.85 < MLD-LBP = 45.08 < MHD-LBP = 48.60) (Figure 3c).

##### 3.2.1.4. MDA levels

The interaction between treatment and operation had a significant effect [F (4, 41) = 2.90, P = 0.03, **ɳ** p2= 0.22]. Although the level of MDA activity in the serum of the subjects in the HD-LBP-treated group in the sham-operated condition was lower than that of those in the OVX condition (MHD-LBP-SHAM = –2.74 < MHD-LBP-OVX = 4.04), the level of MDA activity in the serum of LD-LBP–treated subjects in the sham-operated condition was higher than that of those in the OVX-operated condition (MLD-LBP-SHAM = 5.47 > MLD-LBP-OVX = –1.06) (Figure 3d).

##### 3.2.1.5. 17-β ES measurement

No significant findings were reported in terms of operation and treatment condition or the interaction between these variables.

### 3.3. Immunohistochemistry 

**Figure 4:**
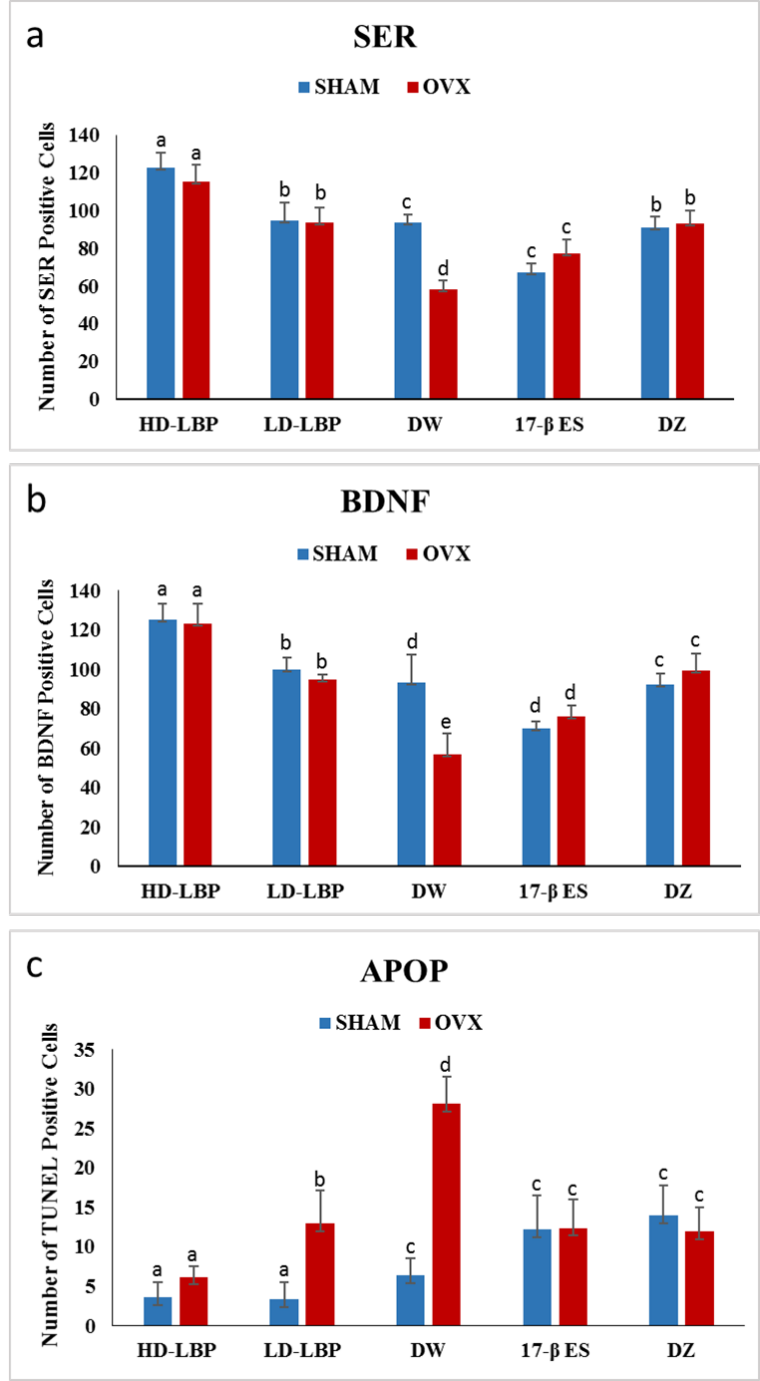
Mean value of the serotonin receptor (SER) (a), BDNF receptor (b), and TUNEL (c) positive cells. Mean-values with the same letters within vertical columns are significantly different (P < 0.05).

The main effect of treatment was significant on SER-positive cell count in the hippocampus region [F (4, 40) = 68.92, P = 0.001, **ɳ** p2 = 0.87], as can be seen in Figure 4a. The hippocampus region of subjects in the HD-LBP-treated groups had a higher count of SER-positive cells than those in the other treated groups. Groups with DW treatment and 17 β-ES treatment had the lowest number of SER-positive cells. The operation had a significant effect on SER-positive cell count in the hippocampus region [F (1, 40) = 10.23, P = 0.003, **ɳ** p2 = 0.20]. Sham-operated groups had a higher number of SER-positive cells than OVX groups (Figure 4a).

Treatment had a significant effect on BDNF positive cell count in the hippocampus region [F (4, 40) = 64.32, P = 0.001, **ɳ** p2 = 0.87]. The hippocampus region of subjects in the HD-LBP-treated groups had a higher number of BDNF-positive cells than those in the other treated groups (Figure 4b). The LD-LBP treatment groups and DZ treatment groups had similar numbers of BDNF-positive cells. The DW treatment and 17 β-ES treatment had similar numbers of BDNF-positive cells, which were also the lowest numbers. The main effect of the operation was significant on BDNF-positive cell count in the hippocampus region [F(1, 40) = 6.84, P = 0.013, **ɳ** p2 = 0.15]. The SHAM groups had a higher number of BDNF-positive cells than OVX- groups. An interaction effect between treatment and operation was significant [F (4, 40) = 11.96, P = 0.001 **ɳ** p2 = 0.54] (Figure 4b). 

Treatment had a significant effect on apoptotic cell count in the hippocampus region [F (4, 40) = 22.86, P = 0.001, **ɳ** p2 = 0.70]. The hippocampus region of subjects in the HD-LBP-treated groups and the LD-LBP-treated groups had a lower apoptotic cell count than the other treated groups (Figure 4c). The DW-treated groups showed the greatest number of TUNEL-positive cells. The operation had a significant effect on the TUNEL-positive cell count in the hippocampus region [F (1, 40) = 52.47, P = 0.001, **ɳ** p2 = 0.57]. SHAM- groups had fewer TUNEL-positive cells than OVX- groups. The effect of interaction between treatment and operation was significant [F (4, 40) = 23.46, P = 0.001 **ɳ** p2 = 0.70] (Figure 4c).

**Figure 5:**
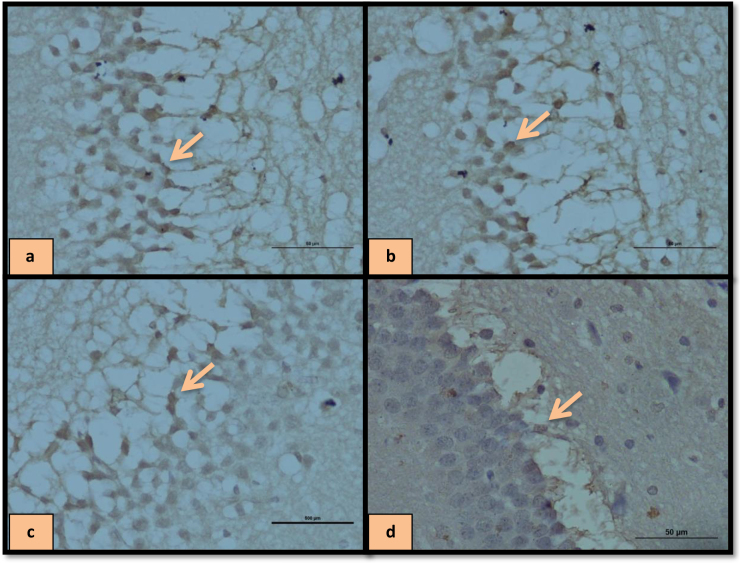
Immune staining of hippocampal SER-positive cells treated with HD-LBP in SHAM group (a), HD-LBP in OVX group (b), DZ in SHAM group (c), DW in SHAM group (d).

**Figure 6:**
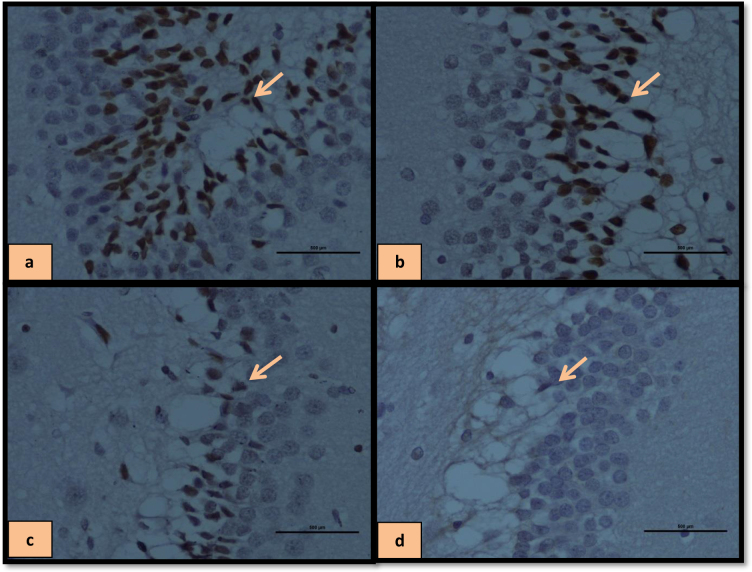
Immune staining of hippocampal BDNF-positive cells treated with HD-LBP in SHAM group (a), HD-LBP in OVX group (b), DZ in SHAM group (c), DW in SHAM group (d).

**Figure 7:**
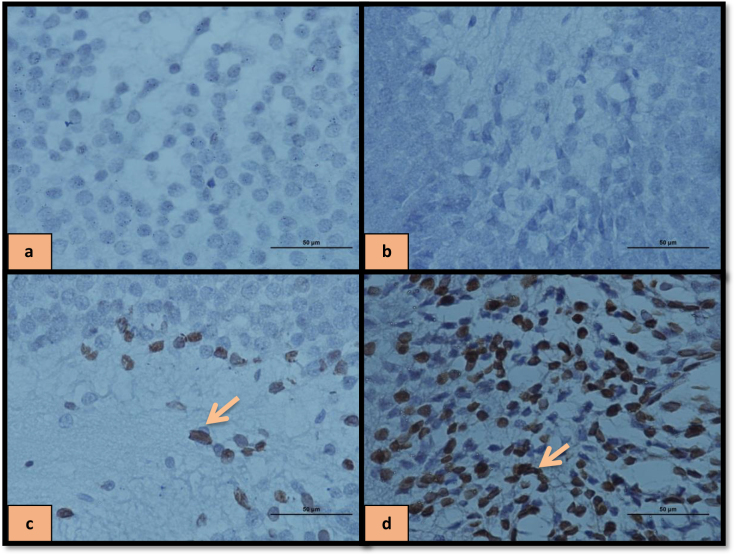
Immune staining of hippocampal TUNEL-positive cells treated with HD-LBP in SHAM group (a), HD-LBP in OVX group (b), DZ in OVX group (c), DW in OVX group (d).

In addition, the interaction between treatment and operation was significant [F (4, 40) = 15.10, P = 0.001 **ɳ** p2 = 0.60]. Immune staining of hippocampal SER-positive cells treated with HD-LBP in the SHAM group (Figure 5a), HD-LBP in the OVX group (Figure 5b), DZ in the SHAM group (Figure 5c),and DW in the SHAM group (Figure 5d) are all shown in Figure 5. Immune staining of hippocampal BDNF-positive cells treated with HD-LBP in the SHAM group (Figure 6a), HD-LBP in the OVX group (Figure 6b), DZ in the SHAM group (Figure 6c), and DW in the SHAM group (Figure 6d) are shown in Figure 6. Immune staining of hippocampal TUNEL-positive cells treated with HD-LBP in the SHAM group (Figure 7a), HD-LBP in the OVX group (Figure 7b), DZ in the OVX group (Figure 7c),and DW in the OVX group (Figure 7d) are shown in Figure 7.

## 4. Discussion

*L. barbarum* fruit, which grows in China, has many beneficial effects, including an anxiolytic effect on human and nonhuman organisms (Gao et al., 2015; Pehlivan Karakaş et al., 2016). Despite the fact that the anxiolytic effect of *L. barbarum* fruit has been attributed to its component polysaccharides in the literature (Gao et al., 2015), no experimental evidence concerning polysaccharides and theeffects on ovariectomized rats has been produced up to now. Our present results provide for the first time evidence that high and low dosages of LBP have effects on the anxious behavior of ovariectomized female rats, and to explain the biochemical and immunohistochemical mechanism of the anxious behavior. 

### 4.1. Behavioral measures

In the present study, ovariectomized and sham-operated female rats were exposed to OFT and EPM for measurement of anxious behavior after various treatment applications. In OFT, decreased time spent in the center, increased time spent at the edge of the open field, and decreased mobility in the open field were accepted as showing high anxiety levels (Benabid et al., 2008). However, in EPM, decreased total distance traveled, increased time spent at the closed arms, decreased time spent at the open arms, mobility, and velocity were accepted as evidence of high anxiety levels (Benabid et al., 2008).These indicators clarify the mechanism of these behaviors.

Our previous research demonstrated that crude methanol extract of *L. barbarum* fruits decreased the anxiety level of rats (Pehlivan Karakaş et al., 2016). Similarly, the present findings showed that LBP treatment decreased the anxiety level of ovariectomized female rats. The subjects in the HD-LBP and LD-LBP treatment groups were more mobile than those in the DW treatment group. These results showed that those in the HD-LBP and LD-LBP reatment groups were less anxious than those in the DW group. Similarly, sham-operated groups were less anxious than OVX-operated groups. Consistent with these findings, there is a shred of evidence that LBP decreased stress-induced anxious behavior in the literature (Gao et al., 2015). Our findings expanded the current knowledge to include the fact that LBP also significantly decreased anxious behavior of ovariectomized rats, which has not been investigated before. The beneficial effect of LPB is evident not only in normal animals but also in ovariectomized ones. In addition to this, a weak or nonsignificant interaction effect between treatment and ovariectomy suggests that the effect of LBP may be relatively independent of the direct effects of gonadal hormones. The plausible role of LBP on anxiety can be made evident via antioxidative stress radicals or enzymes, which were the focus of the current research and are explained in the following sections.

The presence of ovariectomy increases anxiety and the control condition decreases anxiety. Furthermore, LD-LBP and HD-LBP treatments significantly reduced anxious behaviors. On the other hand, 17 *β* -ES and DZ treatments as positive controls showed the same level of anxiety reduction in ovariectomized groups. These results indicate that HD-LBP treatment reduces the anxiolytic effect as much as 17 *β* -ES and DZ treatments in ovariectomized rats. In the literature, Cai et al. (2017) have reported that water extract of *L. barbarum* increased blood serum estrogen levels and uterine size in premature female rats (Cai et al., 2017). In this study, estrogen deficiency depends on ovariectomy, which normally causes anxiety. LPB treatment restores this situation and reduces anxiety. This suggests that LPB can be used for treating the lack of estrogen due to ovariectomy.

In menopause, instead of taking synthetic estrogen and chemical antianxiolytic drugs, natural plant-derived LBP obtained from goji berry fruits may be recommended. In other words, it is wellknown that negative symptoms such as increased anxiety occur during menopause. It is strongly predicted that the use of HD-LBP can treat this adverse effect. In the future, the findings of this study make valuable contributions to medicinal and pharmacological studies in the industrial production and dosing of new plant-derived medicines containing goji berry polysaccharides.

### 4.2. Biochemical measures

Previous research showed that LBP enhanced SOD and GPX enzyme activities; however, it decreased their MDA content (as a marker for oxidative stress). In addition, LBP importantly deactivated caspase-3; thus, LBP may be a hopeful prospect for the treatment of neuronal apoptosis-induced neurodegenerative diseases (Teng et al., 2013). 

Our research also investigated antioxidant enzymes, which may be related to mechanisms for why LBP decreases anxious behaviors. Behavioral disruption could be caused by accumulating oxidative stress in the brain. The oxidative stress level was decreased by increasing antioxidant defense system enzymes such as SOD, CAT, and GPX by decreasing the MDA level. One effective way to decrease oxidative stress may be the implementation of LBP. This was one of our focuses in the present research. Thus, we tested SOD, CAT, GPX, 17 β-ES, and MDA serum concentrations of OVX or sham-operated female rats. We hypothesized that ovariectomy-induced anxious behavior was caused by decreasing antioxidant enzyme activities, so we analyzed antioxidant enzyme levels in the serum of OVX/SHAM female rats after applying behavioral tests. The present biochemical study findings indicated that both HD-LBP and LD-LBP treatments had a greater positive effect on the anxious behavior of rats than other treatments. Moreover, these treatments increased SOD enzyme activities. Our current findings showed that LBP treatments increased the antioxidant defense system. There is some evidence for this finding in the literature that the levels of SOD and CAT enzyme activities were at high concentrations in rats with decreased oxidative stress, but MDA levels were at a low concentration in these rats’ serum (Zhao et al., 2015). In this research, it was expected that 17 β-ES levels in the serum of OVX rats would be lower than those in SHAM groups since 17 β-ES is mostly secreted by ovaries (Cui et al., 2013). This expectation also comes from the recent research evidence indicating that the 17 *β* -ES levels in the serum of group of OVX rats were lower than those in the SHAM group (Han et al., 2015). Similar to this finding, the present research showed that HD-LBP-treated rats had higher SOD activity levels than 17 β-ES treated groups, and that sham-operated groups had higher SOD enzyme activity levels than OVX rats. Taken together, these outcomes showed that HD-LBP treatment had a greater antioxidant effect on ovariectomized female rats than control groups. These findings are in line with those of previous research findings which indicated that LBP application increases SOD activity in rats’ serum levels (Zhao et al., 2015). However, CAT and GPX enzyme activity results showed that 17 β-ES-treated groups had lower CAT and GPX enzyme activities than other groups. The 17 β-ES measurements showed that there were no significant differences in all treatment conditions. These results showed that there was a decreased level of 17 β-ES hormone in the ovariectomized rats’ serum, likely because these rats’ bodies might show resistance to this stress. Another possible reason for this result may be due to less recovery time after the ovariectomy. Another explanation is that the ages of the rats were between 12 or more months. Estrogen is largely produced in the ovarian follicle by conversion of cholesterol to testosterone and androstenedione in the theca internal cells of ovaries, and subsequently, conversion to estrone and estradiol in granulosa cells. Estrogen can also be produced in the corpus luteum and placenta(Berent-Spillsona et al., 2016).

### 4.3. Immunohistochemical measures

Behavioral disruption can be caused by changing levels of neurotransmitters in regions of the brain such as the hippocampus. Ovariectomy causes changing neurotransmitter levels by decreasing estrogen level in serum and some brain regions (Han et al., 2015). Although the fruits of *L. barbarum* were reported in the literature to have a high level of antioxidant activity and to decrease anxious behavior, no research thus far has indicated that it might affect hippocampal neurotransmitter levels such as SER and BDNF or the hippocampal cell apoptosis level of ovariectomized rats. We aimed to explain the effects of LBP on changes in hippocampal neurotransmitters and apoptosis levels of ovariectomized rats. BDNF is a nerve growth factor which plays an important role in the anxious behavior of human and rodents (Duman and Monteggia, 2006). Most studies have shown that decreased anxiety levels were caused by increased BDNF levels in the hippocampus region (Chen et al., 2015). We hypothesized that ovariectomy causes anxious behaviors by decreasing the BDNF level in the hippocampus; LBP treatment might reverse this situation by increasing the BDNF level of the ovariectomized rats. For this purpose, we tested BDNF levels of ovariectomized rats after brain tissues were collected. Our findings showed that HD-LBP treatment groups had higher BDNF levels in the hippocampus region than other treatment groups. The LD-LBP treatment groups had similar BDNF levels as DZ-treated groups; all treatment groups had higher BDNF levels than DW treatment (control) groups. The current findings suggest that increasing BDNF might be caused by activation of gamma-aminobutyric acid (GABA) receptors, because GABA receptors cause increases in both BDNF levels and the levels of its receptors in the hippocampus region. The receptors are tropomyosin receptor kinase B (TrkB) receptors, which play a key role in decreasing anxious behavior (Zhao et al., 2015). Ovariectomy caused increased anxious behavior by decreasing BDNF levels; thus, it might be reversed by downregulation of the BDNF and TrkB pathway. One study also reported that TrkB activation by BDNF stimulated Akt phosphorylation, and that the decreased BDNF level in the hippocampus region of ovariectomized rats might be caused by low activation of the Akt phosphorylation (Rosa et al., 2016).In another study, estrogen has been shown to increase BDNF function through the TrkB receptor, which leads to changes in serotonin circuits that modulate anxious behaviors (Ren-Patterson et al., 2006). Moreover, a recent study showed a positive effect of LBP through myosin light-chain kinase (MLCK)–myosin light chain (MLC) signaling pathway in Caco-2 cells (Li et al., 2020). These pathways suggested that there are alternative ways, even in the intestinal system, that can be mechanisms for the effects of LBP. 

Serotonin and its receptors play an important role in anxious behavior and are responsible for anxiolytic effects. Previous research has indicated that a decrease in SER levels induces anxiety (Rosa et al., 2016). In addition, BDNF is known to protect neuronal cells against oxidative stress (Shin et al., 2019).Although LBP decreases anxiety levels by changing antioxidant biomarkers (Zhao et al., 2015), it is unknown whether or not the anxiolytic effects of LBP on ovariectomized rats are caused by increasing hippocampal SERs. Given this, we aimed to investigate SER in the hippocampus region of ovariectomized rats treated with LBP. Our findings showed that HD-LBP–treatedovariectomized rats had higher SER levels than other treatment groups. This result supported our behavioral results; thus, HD-LBP might produce its anxiolytic effect by increasing the hippocampal SER level. 

Accumulation of reactive oxygen species in the brain causes mitochondrial dysfunction in neuronal cells and increases apoptosis (Shin et al., 2019). It is known that antioxidants decrease the apoptosis level of the rat’s brain (Chen et al., 2018). A previous study also indicated that 20 mg/kg dose of LBP treatment decreased TUNEL-positive neurons in the hippocampus (Wang et al., 2014). Moreover, another study showed that LBP decreased oxidative stress and apoptosis of hippocampal neurons of rats (Zhao et al., 2017). In light of this information, we aimed to analyze whether or not the anxiolytic effect of LBP on ovariectomized rats was due to its reduced effect on apoptotic cells and evaluated TUNEL-positive cell counts in the hippocampus region of OVX and sham-operated rats. In the present study, DW-treated ovariectomized rats had a higher level of TUNEL-positive cell counts than those in sham-operated groups. Moreover, HD-LBP and LD-LBP had more TUNEL-positive neurons than other groups, but HD-LBP treatments were more effective than LD-LBP treatments. These beneficial effects of LBP might be due to its increasing SOD enzyme activity in these groups.

This research investigated for the first time the effects of HD-LBP and LD-LBP obtained from *L. barbarum* fruits on anxiety through behavioral, biochemical, and immunohistochemical means. The results revealed that LD-LBP and HD-LBP treatments decreased anxious behaviors in ovariectomized rats when compared to control groups. The interaction effect also indicated that HD-LBP treatment reduced the anxiolytic effect as much as 17 β-ES and DZ treatments did in ovariectomized rats through bothOFT and EPM testing. Our findings also showed that the HD-LBP treatment decreases anxious behavior by increasing antioxidant enzyme activities, hippocampal SER, and BDNF neurotransmitter levels, and decreasing TUNEL-positive cell counts of ovariectomized rats. In conclusion, given the consideration of new and consistent findings, we suggest that instead of, or in addition to, taking synthetic estrogen and chemical anxiolytic drugs, natural plant-derived LBP obtained from goji berry fruits may be recommended for treating negative symptoms such as increased anxiety during menopause. 
